# Hypersensitivity to betalactam antibiotics: a three years characterization of an Immunoallergy Outpatients' Hospital

**DOI:** 10.1186/2045-7022-4-S3-P67

**Published:** 2014-07-18

**Authors:** Marta Chambel, Inês Mota, Ângela Gaspar, Susana Piedade, Mário Morais-Almeida

**Affiliations:** 1 CUF Descobertas Hospital, Immunoallergy Department, Portugal

## Aim

To characterize the activity developed at our Immunoallergy outpatients' hospital withpatients(patients) referred for suspicion of HS (hypersensitivity) to BL (betalactam antibiotics).

## Methods

Retrospective analysis of clinical files and results of skin testing and DPT (drug provocation tests) from January 2011 to December 2013. All patients were studied according to standardized diagnostic procedures of ENDA/EAACI: serum sIgE (specific IgE) (ImmunoCAP, ThermoFisher) to penicillin G/V, amoxicillin and ampicillin; skin prick and IDT (intradermal tests) to PPL/MD (DAP, Diater), penicillin G, amoxicillin and cefuroxime with immediate and delayed reading. Other penicillin derivatives/cephalosporins were tested if they were the culprit drug. DPT with culprit drug was performed if previous investigation was negative. In confirmed cases an alternative BL was tested.

## Results

224 patients with suspicion of HS to BL were included. Mean age was 34.717yrs; 20% had 18yrs. Female/male ratio was 2.3/1. 11patientshad history of reaction to more than one BL. Penicillins/derivatives were the main culprit drugs (212 patients), mostly amoxicillin (126 patients), 72 in association with clavulanic acid. Cephalosporins were mentioned by 23 pts, namely cefazolin (5 pts). Mucocutaneous manifestations were the most frequent (82%); anaphylaxis occurred in 25 patients, 5 with loss of consciousness. HS to BL was confirmed in 34 patients (15%), all with a DPT negative to an alternative BL. HS was excluded in 156 patients (69,6%); 34 patients are under study. Confirmation of HS was made by: sIgE in 5 patients; skin testing in 23 patients and DPT in 6 patients (amoxicillin-4, clavulanic acid-2). Skin tests were positive to: amoxicillin-15, penicillin-8 (PPL-3), cefazolin-1. Delayed reading of IDT was positive in 3 patients: penicillin-1, amoxicillin-2. A systemic reaction during skin testing occurred in 3 patients: one child with anaphylaxis during IDT with amoxicillin (2.5mg/mL) and 2 adults with urticaria during skin testing with amoxicillin 25mg/mL.

## Conclusions

Investigation of history of HS to BL is of outmost importance because in most of these patients allergological workup is negative and HS to BL is excluded. In most patients with confirmed HS to BL the reaction is IgE-mediated (positive sIgE or immediate positive skin testing); about of HSpatientsseems to have a non-IgE mediated mechanism (positive DPT or positive delayed reading of IDT). Systemic reactions during IDT and DPT also reinforce the need of referral these patients to specialized center.

